# Retrospective validation of an artificial intelligence system for diagnostic assessment of prostate biopsies on the ProMort cohort: study protocol

**DOI:** 10.1136/bmjopen-2025-111361

**Published:** 2025-12-24

**Authors:** Xiaoyi Ji, Renata Zelic, Oskar Aspegren, Nita Mulliqi, Michelangelo Fiorentino, Francesca Giunchi, Luca Molinaro, Sol Erika Boman, Kelvin Szolnoky, Luana Xuan Liu, Andreas Pettersson, Per Henrik Vincent, Martin Eklund, Olof Akre, Kimmo Kartasalo

**Affiliations:** 1 Department of Medical Epidemiology and Biostatistics, Karolinska Institutet, Stockholm, Sweden; 2 Department of Molecular Medicine and Surgery, Karolinska Institutet, Stockholm, Sweden; 3 Department of Pelvic Cancer, Cancer Theme, Karolinska University Hospital, Stockholm, Sweden; 4 Department of Pathology and Cancer Diagnostics, Karolinska University Hospital, Stockholm, Sweden; 5 Department of Medical Epidemiology and Biostatistics, SciLifeLab, Karolinska Institutet, Stockholm, Sweden; 6 Department of Medical and Surgical Sciences, University of Bologna, Bologna, Italy; 7 Department of Pathology, IRCCS Azienda Ospedaliero-Universitaria di Bologna Policlinico di Sant’Orsola, Bologna, Italy; 8 Division of Pathology, AOU Città Della Salute e Della Scienza di Torino, Turin, Italy; 9 Clinical Epidemiology Division, Department of Medicine Solna, Karolinska Institutet, Stockholm, Sweden

**Keywords:** Artificial Intelligence, HISTOPATHOLOGY, Prostate, Retrospective Studies

## Abstract

**Abstract:**

**Introduction:**

Prostate cancer diagnosis and treatment planning depend on accurate histopathological assessment of needle biopsies, particularly through the Gleason scoring system. The inherently subjective nature of the grading creates variability between pathologists, potentially resulting in suboptimal patient management decisions. These reproducibility challenges extend beyond Gleason scoring to encompass other critical diagnostic and prognostic markers, including cancer volume quantification and detection of cribriform morphology patterns and perineural invasion. Artificial intelligence (AI) applications in digital pathology have emerged as promising solutions for enhancing diagnostic consistency and accuracy, with recent research demonstrating that automated systems can match expert-level performance in prostate biopsy evaluation. Nevertheless, comprehensive validation studies have revealed concerning limitations in model generalisability when deployed across different clinical environments and patient populations. Recent systematic reviews revealed widespread risk-of-bias limitations and insufficient external validation in AI diagnostic studies, highlighting critical needs for accumulated evidence supporting generalisability before clinical implementation. Rigorous external validation with preregistered protocols using independent datasets from diverse clinical settings remains essential to establish the reliability and safety of AI-assisted prostate pathology systems.

**Methods and analysis:**

This study protocol establishes a framework for the retrospective external validation of an AI system developed for prostate biopsy assessment, to be conducted on the case-control samples of the National Prostate Cancer Register of Sweden, ProMort study (1998-2015). The primary aim is to evaluate the AI model’s diagnostic accuracy and Gleason grading performance using completely independent datasets separate from any model development or previously used validation cohorts. The diversity of the validation samples, spanning multiple geographic regions, temporal collection periods and reference standards, allows evaluation of model robustness across varied clinical contexts. Secondary aims encompass evaluating AI performance in cancer length estimation and detection of cribriform patterns and perineural invasion. This protocol delineates procedures for data collection, reference standard clarification and prespecified statistical analyses, ensuring comprehensive validation and reliable performance assessment. The study design conforms to established reporting guidelines Checklist for Artificial Intelligence in Medical Imaging (CLAIM) and Standards for Reporting Diagnostic Accuracy Studies using Artificial Intelligence (STARD-AI), and recognised best practices for AI validation in medical imaging.

**Ethics and dissemination:**

Data collection and usage were approved by the Swedish Regional Ethics Review Board and the Swedish Ethical Review Authority (permits 2012/1586-31/1, 2016/613-31/2, 2019-01395, 2019-05220). The study adheres to the Declaration of Helsinki principles, and findings will be made available in open access peer-reviewed publications.

**STRENGTHS AND LIMITATIONS OF THIS STUDY**

- This study incorporates case–control subsamples from Sweden’s largest clinical prostate cancer database (the National Prostate Cancer Register), capturing a broad spectrum of variation across Swedish regions.
- The validation dataset encompasses samples collected from 1998 to 2015, representing one of the first artificial intelligence (AI) validation studies to systematically evaluate performance across such an extensive temporal range, capturing evolving histological sample preparation techniques and changing population characteristics.
- A consistent scanning and annotation platform during digitisation eliminates equipment-related technical variation, while standardised annotation protocols among pathologists ensure traceable and reliable reference standards.
- Case–control design with 50% cancer-related mortality may create spectrum and prevalence bias, limiting comparison with typical clinical populations and other AI studies.
- Differences between the diagnostic reporting guidelines applied to the AI model’s training data and our validation dataset may introduce systematic differences that affect the interpretation of AI-pathologist concordance measurements.

## Introduction

 Systematic histopathological assessment of prostate needle biopsies constitutes an essential component of cancer diagnosis and treatment stratification, fundamentally guiding patient management strategies. The architectural patterns-based Gleason scoring (GS) system, introduced by Gleason and Mellinger,^1^ established the foundational approach for prostate cancer grading using primary and secondary pattern summation (eg, 3+4=7),^2^ though its inherently subjective nature leads to significant interpathologist and intrapathologist variability that places patients at risk of inappropriate treatment decisions.^3 4^ To address these reproducibility concerns, the International Society of Urological Pathology (ISUP) conducted systematic consensus conferences in 2005, 2014 and 2019, progressively refining grading criteria. Notably, major changes to the pattern descriptions and elimination of patterns 1–2 took place in 2005,^5^ followed by introducing the simplified 5-tier Grade Group system in 2014 to replace the complex Gleason score combinations^6^ and establishing refined quantitative assessment criteria in 2019 while including digital pathology workflows combined with artificial intelligence (AI) into consideration for future work.^7^ Similar standardisation challenges have affected the assessment of other clinically relevant histopathological features critical for prognosis and treatment, including cribriform cancer morphology (associated with aggressive behaviour and poor outcomes)^8^ and perineural invasion (PNI, a marker of extraprostatic extension risk).^9^ Additionally, the quantification of cancer extent in prostatic biopsies presents persistent methodological complexity, with recent international surveys revealing that pathologists employ remarkably diverse approaches,^10 11^ including multiple techniques for linear measurements (measuring only the largest focus, summing all foci vs spanning from first to last cancer area). This results in substantial interobserver variability that directly impacts active surveillance eligibility decisions. Despite decades of standardisation efforts through professional societies and evidence-based guideline development, significant diagnostic variability persists, highlighting the critical need for more objective and reproducible approaches to prostate cancer pathological evaluation.

Recent advances in digital pathology and AI have demonstrated remarkable progress in automated prostate cancer diagnosis. Studies by Campanella *et al*,^12^ Ström *et al*^13^ and Bulten *et al*^14^ established that AI systems can achieve pathologist-level diagnostic accuracy. The landmark PANDA Challenge, involving 1290 developers analysing over 10 000 digitised biopsies, achieved agreement of 0.93 (Cohen’s quadratically weighted kappa, QWK) with expert uropathologists on internal validation.^15^ These breakthroughs established a field that has produced multiple Food and Drug Administration (FDA) approved systems including Paige Prostate Detect, which in an evaluation of data from 218 institutions, enabled pathologists to reduce cancer detection errors by 70% compared with unassisted assessment.^16^ The recent emergence of foundation models such as UNI,^17^ Virchow^18 19^ and CONCH,^20^ which are trained on millions of histopathology images in a task-agnostic manner, now provides developers with general-purpose models with minimal fine-tuning requirements for diverse pathology tasks. However, systematic evidence reveals persistent generalisation challenges for AI-assisted prostate biopsy diagnosis. A review investigated 26 regulatory-cleared digital-pathology AI products, among which only 42% had a peer-reviewed external-validation publication.^21^ A 2024 meta-analysis found that 99 out of 100 diagnostic-accuracy studies had at least one high or unclear risk-of-bias or applicability concern.^22^ Performance commonly shows degradation on external cohorts (eg, in PANDA the independent validations achieved agreements of 0.86–0.87, lower than the results on internal validation cohorts), with scanner and stain variability further degrading generalisation.^23 24^ Substantial interobserver variability in Gleason grading and differences in grading conventions introduce label noise that can hinder model transferability.^25 26^ Moreover, temporal domain shifts caused by ageing archived specimens remain largely unexplored in current validation frameworks. This is an important gap, as prognostic applications often rely on historical material spanning decades, and such shifts may substantially affect AI performance across different eras of pathology practice.^27^

Given these challenges, safe clinical deployment of AI-assisted histopathological diagnosis requires robust predeployment evaluation and systematic monitoring in use.^28^ Based on this consideration, we propose an additional retrospective external validation of an in-house, tissue-specific AI system for prostate biopsy assessment, trained end-to-end with the attention-based multiple-instance learning (ABMIL) mechanism for Gleason score classification.^29^ The system was developed on 61,483 WSIs from 4467 patients across four European sites, spanning multiple laboratories, scanners and specimen characteristics.^30^ The results showed that for prostate cancer grading, this task-specific model achieved comparable or superior performance to pipelines based on histopathological foundation models when trained or fine-tuned on the same data. To promote transparency and reproducibility, here we present a prespecified protocol for retrospective external validation of this AI-assisted prostate cancer diagnosis system on the case–control samples of the National Prostate Cancer Register of Sweden (NPCR), ProMort study (1998–2015).^31 32^ We detail objectives, cohort definitions and analysis pipelines to quantify agreement on key diagnostic outputs and to test AI model robustness against major sources of variability. This set-up limits analytic flexibility, reduces post hoc bias and provides an auditable record of the validation workflow to support subsequent clinical evaluation, with the primary aim to technically validate AI performance and to emphasise that caution should be exercised when extrapolating findings to clinical practice.

Robust validation frameworks have emerged to address the rigorous evaluation requirements for AI-based diagnostic and prediction systems in healthcare. Checklist for Artificial Intelligence in Medical Imaging,^33^ updated in 2024, provides specialised reporting requirements for medical imaging AI studies, offering structured guidance covering the complete research pipeline from data acquisition to clinical implementation. The guideline emphasises transparent reporting of image acquisition protocols, reference standard definitions and model evaluation procedures. The STARD-AI (Standards for Reporting Diagnostic Accuracy Studies using Artificial Intelligence) guideline,^34^ updated in 2025, extends the original STARD 2015 framework^35^ with 18 new or modified items specifically addressing AI-centred diagnostic test accuracy studies. The framework was developed through a multistage, multistakeholder consensus process and provides comprehensive reporting recommendations for studies evaluating the diagnostic accuracy of AI-based tests. STARD-AI places particular emphasis on dataset practices including data sources, annotation procedures and partitioning strategies, while addressing critical considerations of algorithmic bias and fairness assessment. The guideline encourages detailed reporting of the AI index test evaluation processes, reference standard methodology and transparent documentation of model performance across different demographic subgroups to ensure equitable healthcare delivery. This retrospective AI validation study adheres to these established reporting frameworks to ensure transparent documentation of data collection procedures, clear definition of reference standard, comprehensive evaluation of AI system performance and robustness, and explicit acknowledgement of study limitations and generalisability considerations.

## Methods and analysis

### Study objectives

The primary objective of this study is to:

- Evaluate agreement between the AI model and pathologists in identifying prostate cancer and assigning Gleason scores in core needle biopsies of the prostate.

In addition, the study addresses three secondary objectives:

- Evaluate the agreement between the AI model and pathologists in measuring the linear extent of cancer (in millimetres) within prostate core needle biopsies.
- Assess the agreement between the AI model and pathologists in identifying PNI in prostate core needle biopsies.
- Assess the agreement between the AI model and pathologists in detecting cribriform patterns of cancer in prostate core needle biopsies.

Clinical implementation, user interaction and the system’s performance when combined with human pathologists are beyond the scope of this study.

### AI system

The AI system to be validated in this study was designed for the histopathological assessment of digitised prostate core needle biopsies.^29^ Based on deep neural networks, the system incorporates specific image preprocessing steps, model architecture and training strategies which were previously optimised during its development phase. The development and initial validation of the AI system followed a protocol-based approach,^30^ with the first peer-reviewed results on its performance recently reported in Blilie *et al*.^36^

#### Model development data

For context, the AI model was originally developed using a large international prostate biopsy dataset comprising 6268 patients and approximately 78 000 whole slide images (WSIs) collected between 2012 and 2023 from 15 clinical sites across 11 countries. The development cohort (n=51 247 WSIs) included material from Capio S:t Göran Hospital, Sweden; Stavanger University Hospital, Norway; and the Swedish STHLM3 trial,^37^ and tuning data (n=1177 WSIs) from Radboud University Medical Centre, The Netherlands; Karolinska University Hospital, Sweden; and the STHLM3 trial were used to assess the generalisation performance of the model prior to design freeze. All data were split at the patient level to prevent any overlap between development, tuning and external validation datasets.

#### System input

The system accepts WSIs in compatible vendor-specific formats, representing formalin-fixed, paraffin-embedded prostate core needle biopsy specimens stained with H&E. Each image may contain one or more tissue sections from one or multiple biopsy cores.

#### System architecture

For inference in validation studies, the AI system uses the fixed architecture of the finalised model. WSIs are first processed with an in-house UNet++^38^-based tissue segmentation model, followed by extraction of 256×256 px patches at 1.0 µm/px from regions containing ≥10% tissue, downsampled from the closest higher-resolution level in the pyramidal image structure. Patches of one WSI are stored as one TFRecord file.^39^ The diagnostic component employs an ABMIL framework^40^ trained with weak slide-level supervision. Patch-level embeddings are extracted using an EfficientNet-V2-S encoder,^41^ producing 1280-dimensional feature vectors. These are processed through a gated-attention ABMIL aggregator, generating a slide-level representation used for classification of the Gleason patterns and associated diagnostic features.

Key hyperparameters include a dropout rate of 0.2 applied to encoder embeddings and intermediate layers, fully connected layers with rectified linear unit (ReLU) activations within the aggregator and classifier, and softmax-normalised attention weights. Comprehensive hyperparameter details are provided in the model development paper.^29^

#### System output

The raw output of the AI system consists of two probability vectors corresponding to the predicted primary and secondary Gleason patterns. Each vector contains four elements, representing the estimated probabilities for the following categories: benign and Gleason grades 3–5.

- Gleason score: The AI system assigns a GS, such as 3+5=8, reflecting the primary and secondary histological patterns identified in the input WSI. The score is derived by selecting the most probable Gleason grade from each of the two prediction vectors. Possible scores range from 3+3=6 (least aggressive) to 5+5=10 (most aggressive), with 0+0 representing benign samples.
- ISUP grade: The assigned GS is further mapped to an ISUP grade from 1 to 5, which categorises cancer aggressiveness on an ordinal scale: ISUP 1 (GS 6), ISUP 2 (GS 3+4=7), ISUP 3 (GS 4+3=7), ISUP 4 (GS 8) and ISUP 5 (GS 9–10). Benign cases are assigned an ISUP grade of 0.
- Cancer diagnosis: A binary outcome is determined from the predicted ISUP grade, with samples assigned an ISUP grade >0 classified as cancer-positive by the system. In addition, the system outputs a continuous probability estimate of cancer presence, calculated as one minus the minimum predicted probability of the benign class across the two Gleason pattern outputs.
- Cancer extent: The system will estimate the cancer length within the WSI in millimetres, providing a quantitative measure of the tumour’s spatial extent in the tissue.
- Cribriform cancer: A probability predicting the presence of cribriform morphology within the sample will be reported.
- PNI: A probability predicting the presence of PNI within the sample will be reported.
- Visualisation: Prediction results can be presented as visual overlays on the WSI, indicating regions associated with specific Gleason patterns, cribriform morphology and PNI, with the exact appearance and format determined by subsequent processing.

### Study design

In this study, we will perform a fully external validation of the diagnostic performance of the AI system, using retrospectively collected prostate biopsy data from a sample of Swedish men diagnosed with prostate cancer from 1998 to 2015 as a part of the ProMort I and ProMort II studies. The dataset represents a heterogeneous clinical environment, comprising patients from diverse backgrounds and clinical stages from multiple regions across Sweden, drawn from individuals whose samples were not involved in any phase of model development. The samples were digitised with a scanner of a different model (3DHistech) than the AI training data (Aperio, Hamamatsu, Philips) and prepared in laboratories not included in the development set. This introduces a domain shift in sample preparation and image acquisition characteristics, thereby providing a test of the AI system’s generalisability to previously unseen laboratories and scanner hardware.

Interobserver variability among pathologists introduces heterogeneity in the reference standards within the validation dataset, as different pathologists independently annotated overlapping but non-identical subsets of cases. Because sample assignments were not stratified by cohort or region, variation in annotations arises in a non-systematic manner, which complicates the interpretation of the AI system’s performance. Observed differences in performance may thus reflect not only the model’s ability to generalise across diverse clinical data, but also inconsistencies in the human reference annotations. These two sources of variation, model generalisation and annotation variability, are difficult to disentangle and cannot be quantified. To address this challenge, we designed the validation study using two complementary strategies. First, we selected the annotations from a single pathologist, whose assessments cover the majority of the validation samples, as the primary reference standard. This enables consistent benchmarking across clinical sites and supports the evaluation of technical generalisation performance without confounding effects from interobserver variation, sample origin or collection time. Second, for subsets of the ProMort I and ProMort II cohorts that were independently reviewed by multiple pathologists, we explicitly compared interobserver variability with AI-pathologist agreement. This allows us to contextualise the AI system’s performance relative to human variability and assess whether the AI demonstrates a comparable or higher level of consistency across diverse clinical settings.

To ensure the integrity of the evaluation, the model development and validation phases were strictly separated to prevent any risk of information leakage. The study uses digitised images of core needle biopsies from prostate cancer patients where both the AI system and human pathologists independently assessed the biopsy samples, with no access to each other’s results, guaranteeing a blinded evaluation process. No adjustments or modifications to any model parameters or settings were conducted during the validation procedure.

The retrospective nature of this validation study does not inherently affect the validity of agreement metrics between AI and pathologists. However, retrospective designs lack real-time diagnostic feedback, precluding additional investigations (eg, step-sections, immunohistochemistry) that would typically be employed to resolve diagnostic ambiguities in clinical practice. While cases with substantial AI-pathologist discrepancies are planned to undergo independent expert re-evaluation, this assessment remains constrained to existing histological material, potentially limiting the interpretation of apparent AI 'errors’ that might be resolved through additional clinical workup.

Clinical and pathological characteristics of the ProMort cohorts are presented in tables1  2. Annotation coverage by each pathologist for diagnostic parameters is also summarised for Gleason grades and cancer length in table 3, and for cribriform morphology and PNI in table 4. These pathologist annotations constitute the reference standards for AI performance evaluation.

**Table 1:** Patient-level clinical and pathological characteristics of ProMort I sample at the time of diagnosis from NPCR

Descriptives	All subjects				Scanned subjects				AI validation subjects			
	Case		Control		Case		Control		Case		Control	
	N	%	N	%	N	%	N	%	N	%	N	%
N	1710	–	1710	–	1178	–	1113	–	146	–	144	–
Age, years (mean, SD)	74	7.76	68	7.77	73	7.77	67	7.53	73	7.60	67	6.20
Age, years (median, IQR)	74	69.00, 79.00	68	62.00, 73.00	74	68.00, 79.00	67	62.00, 72.00	74	69.00, 78.00	67	63.50, 72.00
PSA, ng_mL(mean, SD)	10	4.58	9	4.29	11	4.39	9	4.19	10	4.35	8	3.88
PSA, ng_mL (median, IQR)	10	6.90, 14.00	8	5.60, 11.30	10	7.20, 14.00	8	6.00, 12.00	10	6.80, 13.80	8	5.55, 10.10

County of residence at diagnosis												
Blekinge	22	1.29	22	1.29	13	1.10	14	1.26	0	0.00	0	0.00
Dalarna	52	3.04	52	3.04	35	2.97	37	3.32	0	0.00	0	0.00
Gävleborg	42	2.46	42	2.46	32	2.72	32	2.88	0	0.00	0	0.00
Gotland	3	0.18	3	0.18	0	0.00	0	0.00	0	0.00	0	0.00
Halland	67	3.92	67	3.92	54	4.58	50	4.49	0	0.00	0	0.00
Jämtland	26	1.52	26	1.52	0	0.00	0	0.00	0	0.00	0	0.00
Jönköping	95	5.56	95	5.56	73	6.20	80	7.19	0	0.00	0	0.00
Kalmar	50	2.92	50	2.92	27	2.29	24	2.16	0	0.00	0	0.00
Kronoberg	31	1.81	31	1.81	13	1.10	21	1.89	0	0.00	0	0.00
Norrbotten	32	1.87	32	1.87	17	1.44	20	1.80	0	0.00	0	0.00
Skåne	227	13.27	227	13.27	160	13.58	152	13.66	138	94.52	128	88.89
Södermanland	44	2.57	44	2.57	38	3.23	38	3.41	0	0.00	0	0.00
Stockholm	209	12.22	209	12.22	141	11.97	135	12.13	0	0.00	0	0.00
Uppsala	59	3.45	59	3.45	41	3.48	34	3.05	0	0.00	0	0.00
Värmland	42	2.46	42	2.46	34	2.89	34	3.05	0	0.00	0	0.00
Västerbotten	49	2.87	49	2.87	35	2.97	27	2.43	0	0.00	0	0.00
Västernorrland	52	3.04	52	3.04	40	3.40	43	3.86	0	0.00	0	0.00
Västmanland	38	2.22	38	2.22	30	2.55	27	2.43	0	0.00	0	0.00
Västra Götaland	439	25.67	439	25.67	315	26.74	264	23.72	0	0.00	0	0.00
Örebro	42	2.46	42	2.46	19	1.61	25	2.25	8	5.48	16	11.11
Östergötland	89	5.20	89	5.20	61	5.18	56	5.03	0	0.00	0	0.00

Year of diagnosis												
1998	176	10.29	176	10.29	112	9.51	94	8.45	22	15.07	17	11.81
1999	195	11.40	195	11.40	117	9.93	117	10.51	17	11.64	17	11.81
2000	207	12.11	207	12.11	137	11.63	123	11.05	19	13.01	23	15.97
2001	199	11.64	199	11.64	140	11.88	131	11.77	24	16.44	22	15.28
2002	206	12.05	206	12.05	145	12.31	130	11.68	9	6.16	11	7.64
2003	173	10.12	173	10.12	131	11.12	117	10.51	14	9.59	11	7.64
2004	163	9.53	163	9.53	124	10.53	114	10.24	12	8.22	14	9.72
2005	126	7.37	126	7.37	84	7.13	98	8.81	11	7.53	13	9.03
2006	91	5.32	91	5.32	69	5.86	60	5.39	5	3.42	4	2.78
2007	65	3.80	65	3.80	49	4.16	48	4.31	4	2.74	5	3.47
2008	52	3.04	52	3.04	33	2.80	35	3.14	6	4.11	5	3.47
2009	40	2.34	40	2.34	27	2.29	32	2.88	2	1.37	1	0.69
2010	12	0.70	12	0.70	7	0.59	10	0.90	0	0.00	0	0.00
2011	5	0.29	5	0.29	3	0.25	4	0.36	1	0.68	1	0.69

Clinical tumour stage at diagnosis												
T1	2	0.12	2	0.12	1	0.08	1	0.09	0	0.00	0	0.00
T1a	75	4.39	119	6.96	6	0.51	14	1.26	1	0.68	5	3.47
T1b	92	5.38	51	2.98	8	0.68	6	0.54	2	1.37	1	0.69
T1c	521	30.47	854	49.94	397	33.70	622	55.88	56	38.36	83	57.64
T2	1020	59.65	684	40.00	766	65.03	470	42.23	87	59.59	55	38.19

Diagnostic primary Gleason pattern												
1	6	0.35	9	0.53	2	0.17	6	0.54	2	1.37	0	0.00
2	89	5.20	132	7.72	51	4.33	73	6.56	11	7.53	14	9.72
3	653	38.19	781	45.67	489	41.51	567	50.94	69	47.26	83	57.64
4	236	13.80	81	4.74	170	14.43	57	5.12	20	13.70	7	4.86
5	1	0.06	0	0.00	1	0.08	0	0.00	0	0.00	0	0.00
Missing	725	42.40	707	41.35	465	39.47	410	36.84	44	30.14	40	27.78

Diagnostic secondary Gleason pattern												
1	0	0.00	4	0.23	0	0.00	3	0.27	0	0.00	0	0.00
2	81	4.74	125	7.31	47	3.99	82	7.37	9	6.16	10	6.94
3	598	34.97	682	39.88	434	36.84	480	43.13	56	38.36	74	51.39
4	302	17.66	183	10.70	230	19.52	133	11.95	37	25.34	18	12.50
5	1	0.06	0	0.00	1	0.08	0	0.00	0	0.00	0	0.00
Missing	728	42.57	716	41.87	466	39.56	415	37.29	44	30.14	42	29.17

Diagnostic Gleason score												
2	9	0.53	11	0.64	6	0.51	5	0.45	2	1.37	1	0.69
3	23	1.35	28	1.64	17	1.44	19	1.71	9	6.16	4	2.78
4	75	4.39	104	6.08	41	3.48	60	5.39	10	6.85	11	7.64
5	149	8.71	250	14.62	99	8.40	165	14.82	12	8.22	23	15.97
6	531	31.05	810	47.37	388	32.94	549	49.33	42	28.77	64	44.44
7	783	45.79	382	22.34	585	49.66	263	23.63	59	40.41	29	20.14
Missing	140	8.19	125	7.31	42	3.57	52	4.67	12	8.22	12	8.33

Diagnosis established by												
Only clinical diagnosis	4	0.23	1	0.06	1	0.08	1	0.09	0	0.00	1	0.69
Histopathology	1584	92.63	1624	94.97	1149	97.54	1082	97.21	145	99.32	143	99.31
Cytology	73	4.27	36	2.11	3	0.25	4	0.36	0	0.00	0	0.00
Missing	49	2.87	49	2.87	25	2.12	26	2.34	1	0.68	0	0.00

Primary treatment												
Conservative	785	45.91	648	37.89	494	41.94	368	33.06	50	34.25	38	26.39
Curative	407	23.80	849	49.65	308	26.15	607	54.54	44	30.14	93	64.58
Non-curative	489	28.60	185	10.82	354	30.05	120	10.78	49	33.56	13	9.03
Missing	29	1.70	28	1.64	22	1.87	18	1.62	3	2.05	0	0.00

Data are presented for (1) all patients from the original case-control study design (N=3420), (2) patients with successfully scanned tissue biopsies (N=2291) and (3) patients included for AI system validation after applying exclusion criteria on the subjects re-reviewed in 2017 (N=290). All clinical and pathological information is derived from the original NPCR database, with ‘missing’ indicating that information on variables recorded at diagnosis was not available in the underlying cohort.

NPCR, National Prostate Cancer Register; PSA, prostate-specific antigen.

**Table 2:** Patient-level clinical and pathological characteristics of ProMort II sample at the time of diagnosis from NPCR

Descriptives	All subjects				Scanned subjects				AI validation subjects			
	Case		Control		Case		Control		Case		Control	
	N	%	N	%	N	%	N	%	N	%	N	%
N	500	–	500	–	405	–	429	–	373	–	374	–
Age, years (mean, SD)	75	8.11	69	8.30	405	–	429	–	74	8.02	68	8.43
Age, years (median, IQR)	75	70.00, 81.00	69	62.00, 75.00	75	70.00, 80.00	68	62.00, 75.00	75	69.00, 80.00	68	62.00, 75.00
PSA, ng/mL (mean, SD)	104	499.88	29	86.94	75	70.00, 80.00	68	62.00, 75.00	107	561.93	29	95.71
PSA, ng/mL (median, IQR)	26	12.00, 65.00	11	6.00, 21.00	26	12.00, 65.00	11	5.80, 20.00	26	12.00, 65.00	11	6.10, 20.00
PSA missing	10	2.00	8	1.60	8	1.98	5	1.18	5	1.34	1	0.27

County of residence at diagnosis												
Dalarna	45	9.00	45	9.00	36	8.89	41	9.56	35	9.38	37	9.89
Gävleborg	46	9.20	46	9.20	38	9.38	39	9.09	36	9.65	34	9.09
Halland	44	8.80	44	8.80	40	9.88	39	9.09	35	9.38	36	9.63
Jönköping	40	8.00	40	8.00	34	8.40	34	7.93	30	8.04	31	8.29
Kalmar	28	5.60	28	5.60	27	6.67	26	6.06	25	6.70	24	6.42
Kronoberg	19	3.80	19	3.80	11	2.72	13	3.03	9	2.41	11	2.94
Norrbotten	32	6.40	32	6.40	27	6.67	20	4.66	22	5.90	17	4.55
Skåne	128	25.60	128	25.60	113	27.90	114	26.57	109	29.22	96	25.67
Värmland	43	8.60	43	8.60	32	7.90	37	8.62	31	8.31	35	9.26
Västmanland	33	6.60	33	6.60	27	6.67	32	7.46	27	7.24	26	6.95
Örebro	42	8.40	42	8.40	20	4.94	34	7.93	14	3.75	27	7.22

Year of diagnosis												
1998	51	10.20	51	10.20	33	8.15	37	8.62	29	7.77	26	6.95
1999	52	10.40	52	10.40	34	8.40	40	9.32	32	8.58	31	8.29
2000	39	7.80	39	7.80	29	7.16	29	6.76	28	7.51	22	6.88
2001	43	8.60	43	8.60	38	9.38	38	8.86	34	9.12	36	9.63
2002	43	8.60	43	8.60	34	8.40	40	9.32	29	7.77	38	10.16
2003	48	9.60	48	9.60	41	10.12	43	10.02	39	10.46	37	9.89
2004	42	8.40	42	8.40	39	9.63	38	8.86	36	9.65	35	9.36
2005	39	7.80	39	7.80	35	8.64	35	8.16	32	8.58	28	7.49
2006	24	4.80	24	4.80	19	4.69	21	4.90	17	4.56	18	4.81
2007	18	3.60	18	3.60	17	4.20	16	3.73	16	4.29	16	4.28
2008	30	6.00	30	6.00	23	5.68	27	6.29	21	5.63	25	6.68
2009	28	5.60	28	5.60	25	6.17	26	6.06	22	5.90	24	6.42
2010	13	2.60	13	2.60	11	2.72	10	2.33	11	2.95	10	2.67
2011	16	3.20	16	3.20	16	3.95	15	3.50	16	4.29	15	4.01
2012	4	0.80	4	0.80	3	0.74	4	0.93	3	0.80	3	0.80
2013	6	1.20	6	1.20	5	1.23	6	1.40	5	1.34	6	1.60
2014	3	0.60	3	0.60	2	0.49	3	0.70	2	0.54	3	0.80
2015	1	0.20	1	0.20	1	0.25	1	0.23	1	0.27	1	0.27

Clinical tumour stage at diagnosis												
T0	1	0.20	4	0.80	1	0.25	3	0.70	0	0.00	2	0.53
T1a	4	0.80	20	4.00	2	0.49	14	3.26	1	0.27	1	0.27
T1b	11	2.20	14	2.80	7	1.73	13	3.03	0	0.00	0	0.00
T1c	62	12.40	170	34.00	55	13.58	156	36.36	52	13.94	145	38.77
T2	136	27.20	180	36.00	115	28.40	151	35.20	107	28.69	140	37.43
T3	229	45.80	94	18.80	186	45.93	79	18.41	177	47.45	74	19.79
T4	48	9.60	12	2.40	34	8.40	9	2.10	32	8.58	9	2.41
TX	9	1.80	6	1.20	5	1.23	4	0.93	4	1.07	3	0.80
Missing	1	0.20	0	0.00	1	0.25	0	0.00	1	0.27	0	0.00

N-stage at diagnosis												
N0	36	7.20	82	16.40	30	7.41	77	17.95	27	7.24	63	16.84
N1	25	5.00	9	1.80	19	4.69	8	1.86	17	4.56	6	1.60
NX	438	87.60	409	81.80	355	87.65	344	80.19	328	87.94	305	81.55
M0	203	40.60	223	44.60	168	41.48	192	44.76	157	42.09	167	44.65
MX	296	59.20	276	55.20	236	58.27	236	55.01	215	57.64	206	55.08
Missing	1	0.20	1	0.20	1	0.25	1	0.23	1	0.27	1	0.27

Diagnostic primary Gleason pattern												
1	0	0.00	1	0.20	0	0.00	1	0.23	0	0.00	1	0.27
2	9	1.80	32	6.40	9	2.22	29	6.76	7	1.88	24	6.42
3	108	21.60	251	50.20	100	24.69	234	54.55	94	25.20	209	55.88
4	192	38.40	73	14.60	180	44.44	66	15.38	172	46.11	64	17.11
5	33	6.60	7	1.40	29	7.16	7	1.63	25	6.70	6	1.60
Missing	158	31.60	136	27.20	87	21.48	92	21.45	75	20.11	70	18.72

Diagnostic secondary Gleason pattern												
2	9	1.80	31	6.20	8	1.98	30	6.99	7	1.88	22	5.88
3	117	23.40	210	42.00	111	27.41	194	45.22	102	27.35	177	47.33
4	123	24.60	103	20.60	114	28.15	93	21.68	110	29.49	87	23.26
5	90	18.00	17	3.40	82	20.25	17	3.96	76	20.38	16	4.28
Missing	161	32.20	139	27.80	90	22.22	95	22.14	78	20.91	72	19.25

Diagnostic Gleason score												
2	0	0.00	1	0.20	0	0.00	1	0.23	0	0.00	1	0.27
3	1	0.20	4	0.80	1	0.25	4	0.93	1	0.27	3	0.80
4	5	1.00	10	2.00	5	1.23	9	2.10	4	1.07	7	1.87
5	15	3.00	67	13.40	14	3.46	61	14.22	10	2.68	47	12.57
6	64	12.80	171	34.20	59	14.57	159	37.06	54	14.48	142	37.97
7	129	25.80	123	24.60	119	29.38	115	26.81	114	30.56	108	28.88
8	74	14.80	32	6.40	68	16.79	27	6.29	64	17.16	23	6.15
9	102	20.40	15	3.00	92	22.72	15	3.50	84	22.52	14	3.74
10	12	2.40	6	1.20	10	2.47	6	1.40	9	2.41	5	1.34
Missing	98	19.60	71	14.20	37	9.14	32	7.46	33	8.85	24	6.42

Primary reason for cancer diagnosis												
Health examination (PSA)	69	13.80	138	27.60	64	15.80	122	28.44	59	15.82	115	30.75
LUTS	133	26.60	106	21.20	116	28.64	91	21.21	105	28.15	81	21.66
Other symptoms	193	38.60	154	30.80	156	38.52	138	32.17	146	39.14	119	31.82
Missing	105	21.00	102	20.40	69	17.04	78	18.18	63	16.89	59	15.78

Primary treatment												
Conservative	83	16.60	149	29.80	67	16.54	120	27.97	59	15.82	92	24.60
Curative	50	10.00	206	41.20	42	10.37	188	43.82	40	10.72	171	45.72
Non-curative	361	72.20	140	28.00	292	72.10	118	27.51	270	72.39	109	28.88
Dead before treatment decision	4	0.80	0	0.00	2	0.49	0	0.00	2	0.54	0	0.00
Missing	2	0.40	5	1.00	2	0.49	3	0.70	2	0.54	3	0.80

Data are presented for (1) all patients from the original case–control study design (N=1000), (2) patients with successfully scanned tissue biopsies (N=834, including four duplicate controls that were subsequently removed) and (3) patients included for AI system validation after applying exclusion criteria on the subjects re-reviewed in 2019 (N=747). All clinical and pathological information is derived from the original NPCR database, with ‘missing’ indicating that information on variables recorded at diagnosis was not available in the underlying cohort.

.LUTS, lower urinary tract symptoms; NPCR, National Prostate Cancer Register; PSA, prostate-specific antigen.

**Table 3:** Reference standard protocols with respect to Gleason grading and cancer length estimation for cases for AI model validation

Data source		Patient number	Slide number	Core number	Outcomes of interest	Reviewer	Annotated cores
**ProMort I**	Skåne and Örebro	290	1780	2096	Gleason score	FG	2093
						LM	908
						MF	558
					Cancer length	FG	2093
**ProMort II**	Case–control	693	3862	8242	Gleason score	FG	7903
						MF	339
					Cancer length	FG	7903
						MF	339
	Software validation	54	322	374	Gleason score	FG	373
						MF	372
						OAs	352
					Cancer length	FG	373
						MF	372
						OAs	352

ProMort II comprises two subsamples: case–control and software validation. All annotations were performed at the core level, including both cancer-positive and cancer-negative cores. In the reviewer column, each row represents an individual pathologist with independent assessments in a blinded manner.

AI, artificial intelligence.

**Table 4:** Reference standard protocols with respect to cribriform pattern and PNI detection for cases for AI model validation

Data source		Patient number	Core number	Focus region number	Reviewer	Annotated cores	Annotated focus regions
**ProMort I**	Skåne and Örebro	273	915	2394	FG	911	2381
					LM	483	1261
					MF	557	1397
**ProMort II**	Case–control	672	3323	5954	FG	2988	5587
					MF	335	368
	Software validation	52	230	895	FG	228	496
					MF	218	320
					OAs	204	78

ProMort II comprises two subsamples: case–control and software validation. All annotations were performed at the focus region level and aggregated to core level for validation. In the focus region number column, the counts include only the regions with tumours. In the reviewer column, each row represents an individual pathologist with independent assessments in a blinded manner.

AI, artificial intelligence; PNI, perineural invasion.

### Data sources

#### ProMort I

##### Source

ProMort I is a nested case–control study derived from NPCR.^31^ Men diagnosed with low-risk to intermediate-risk prostate cancer between 1 January 1998 and 31 December 2011, were eligible for inclusion. Risk stratification criteria included: clinical stage T1-T2, GS≤7 or WHO grade 1 if GS unavailable, serum Prostate-Specific Antigen (PSA) <20 ng/mL, and absence of nodal (N0/Nx) or distant (M0/Mx) metastases. From approximately 58 000 eligible men, 1735 prostate cancer deaths were identified through 31 December 2012. Controls were selected through incidence-density sampling, matched 1:1 on year and institution of diagnosis. After excluding 25 cases lacking eligible controls, the final cohort comprised 1710 matched case–control pairs.

##### Slide digitisation and histopathological review

Diagnostic specimens were retrieved from pathology departments throughout Sweden and digitised at Örebro University Hospital between November 2015 and February 2016 using a Pannoramic 250 Flash II scanner (3DHistech, Budapest, Hungary) at 40×magnification (0.19 µm/pixel resolution). From 3420 sampled patients, slides were successfully retrieved and scanned for 2290 patients (67%), yielding 14 036 digital images stored in MRXS format.

A subset of 356 patients from Örebro (n=44) and Skåne (n=312) counties was selected as a pathological re-review subsample to confirm low-risk to intermediate-risk classification and establish interobserver concordance. The review was conducted between June 2017 and April 2018, with annotation terminated after 313 patients (44 from Örebro, 269 from Skåne; 159 cases, 154 controls) based on interim assessment of data adequacy.

##### Reference standard protocol

Digital assessment was performed using a virtual microscopy system developed in collaboration between the Centre for Advanced Studies, Research and Development in Sardinia (CRS4), Pula, Italy and the ProMort study.^42^ Three genitourinary pathologists (FG, LM and MF with 8, 4 and 19 years of experience in genitourinary pathology at the time of the review, respectively) evaluated cases following the 2014 ISUP modified Gleason grading system. The pathologists were blinded to the original clinical and histopathological information and the case–control status. The annotation followed a structured workflow:

- **Slide-level screening:** First, FG systematically reviewed all slides for each patient, first determining whether slides should be excluded by rejecting duplicate slides, slides lacking prostatic tissue or specimens other than core-needle biopsies (eg, TURP specimens, lymph node specimens). For eligible slides, FG identified all tissues at the core level with spatial delineation. To avoid redundancy, when cores were represented by multiple slices, only the most representative slice was selected for subsequent scoring.
- **Core and focus region annotation:**
  - FG performed comprehensive annotation including cancer detection, tumour length measurement and Gleason grading for each core. For cancer positive cores, all areas with cancer were marked as separate focus regions with spatial delineation, plus one selected area of normal tissue per core. Each positive focus region underwent detailed prognostic feature assessment, including perineural involvement and cribriform pattern.
  - Then LM independently reviewed all cancer-positive cores annotated by FG for Gleason grading and additionally assessed prognostic features for all positive focus regions within a randomly selected subset of these cores.
  - Cases with interobserver disagreement on Gleason grading underwent adjudication by MF, who reviewed both Gleason grading and prognostic features for positive focus regions within the discordant cores.

**Figure 1:**
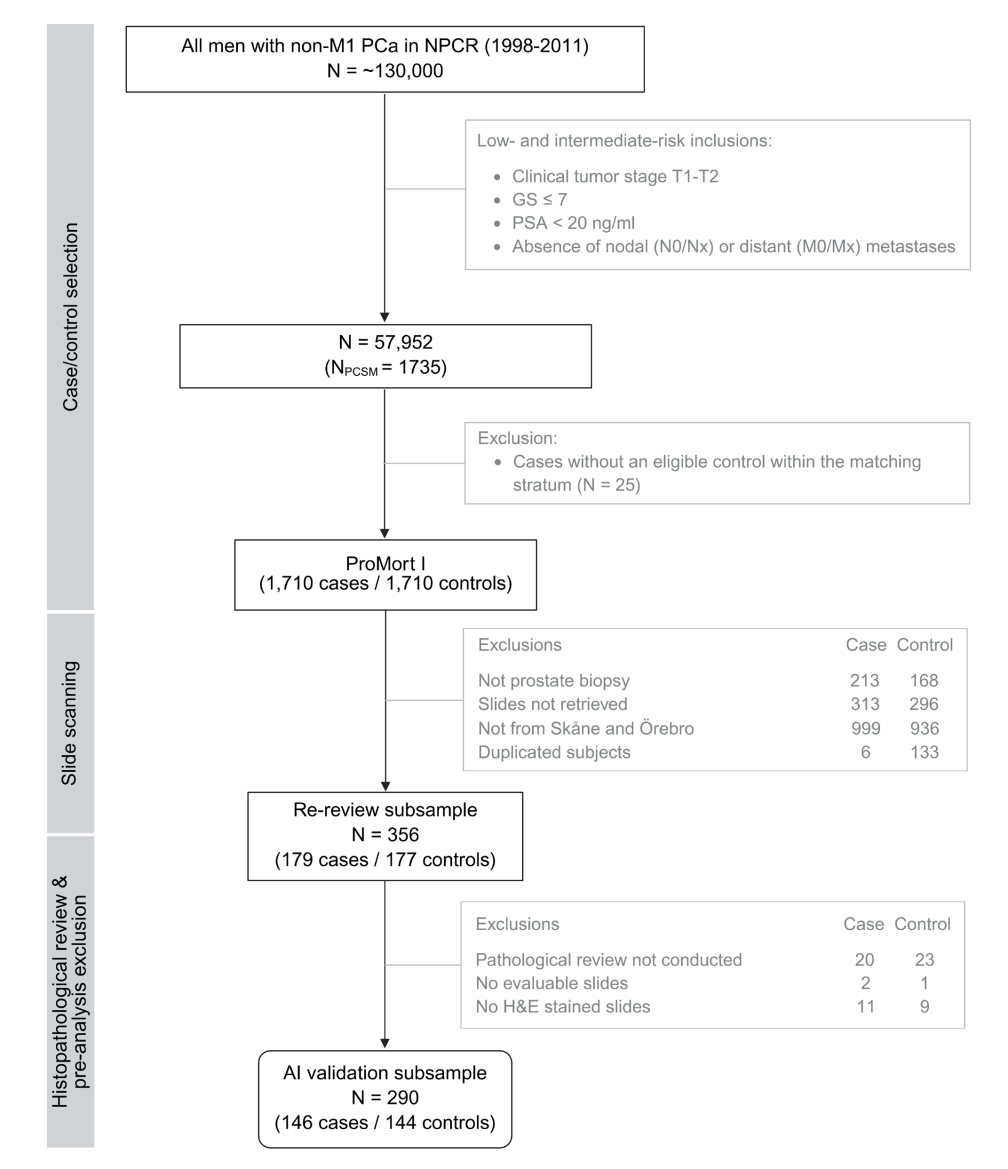
Flow chart of population selection for ProMort I sample, its re-review subsample and AI validation subsample. N represents the number of patients. GS, Gleason score; M, metastasis; N, nodes; NPCR, National Prostate Cancer Register of Sweden; PCa, prostate cancer; PCSM, prostate cancer-specific mortality; PSA, prostate-specific antigen; T, tumour

Ultimately, 290 patients (146 cases and 144 controls) were used for the AI model validation. A detailed description of the ProMort I inclusion and exclusions is presented in a flow chart in figure 1. Population characteristics at the time of diagnosis are summarised in table 1. The annotation protocol is available in online supplemental material 1.

### ProMort II

#### Source

ProMort II is a case–control sample of men in NPCR diagnosed with non-metastatic (M0/Mx) prostate cancer between 1 January 1998 and 31 December 2015.^32^ The sample was selected from 11 of 21 Swedish counties, chosen based on their likelihood of providing slides for digitisation. The case–control sample comprised 500 cases (random sample of men who died of prostate cancer) and 500 controls (sampled with replacement by incidence density sampling), matched 1:1 on year and county of diagnosis.

#### Slide digitisation and histopathological review

Biopsy slides were retrieved from pathology departments and scanned at Örebro University Hospital using the same scanner and scanning protocol as ProMort I. The scanning took place between May 2017 and January 2018, approximately 2–20 years after slide preparation. From the 1000 sampled patients, slides were successfully retrieved and scanned for 830 patients (83%), yielding 5536 digital images stored in MRXS format.

Two complementary subsamples were then defined:

- **Software validation subsample** (n=60): A random sample of 26 cases and 34 controls from two counties (Örebro n=25, Värmland n=35) selected for multipathologist annotation, with the original purpose to assess interobserver variability based on different microscopy setups.^25^
- **Case–control subsample** (n=714): The remaining subjects with evaluable slides after extraction of the software validation set, with 359 cases and 355 controls.

#### Reference standard protocol

Digital assessment used the same virtual microscopy platform as described for ProMort I. Three genitourinary pathologists (FG, MF and OAs with 10, 21 and 2 years of experience at the time of the review, respectively) were involved in independently reviewing the scanned images following the 2016 WHO Classification of Tumours of the Urinary System and Male Genital Organs. The pathologists were blinded to the original clinical and histopathological information and the case–control status.

The annotation workflow followed the same structured approach as ProMort I, consisting of slide-level screening and core and focus region annotation. However, the delineation process differed between subsamples. In the software validation subsample, each pathologist independently performed slide-level screening and manually delineated their own cores and focus regions. In the case–control subsample, initial tissue detection and core delineation were generated automatically by the digital assessment platform used in the ProMort project. The automated procedure occasionally partitioned single biological biopsy cores into multiple tissue fragments in the presence of gaps or discontinuities. Pathologist FG performed manual corrections, although not all instances were adjusted. Each delineated fragment was treated as a core for subsequent annotation. For each slide assigned to a pathologist, the reviewer first determined whether it should be excluded by rejecting duplicate slides, slides lacking prostatic tissue or specimens other than core-needle biopsies (eg, TURP specimens, lymph node specimens). To enable fair interobserver comparisons at the core level, annotations were subsequently aligned to match corresponding anatomical regions across different pathologists’ markings.

- **Case–control subsample:** Each core was reviewed only once by a single pathologist (FG or MF), including cancer detection, tumour length measurement, Gleason grading and prognostic feature assessment, including perineural involvement and cribriform pattern.
- **Software validation subsample:** Each core was first reviewed three times: twice by FG and once by MF. In 2020, OAs reviewed all 60 patients, applying a workflow following the same annotation protocol as FG and MF.

**Figure 2:**
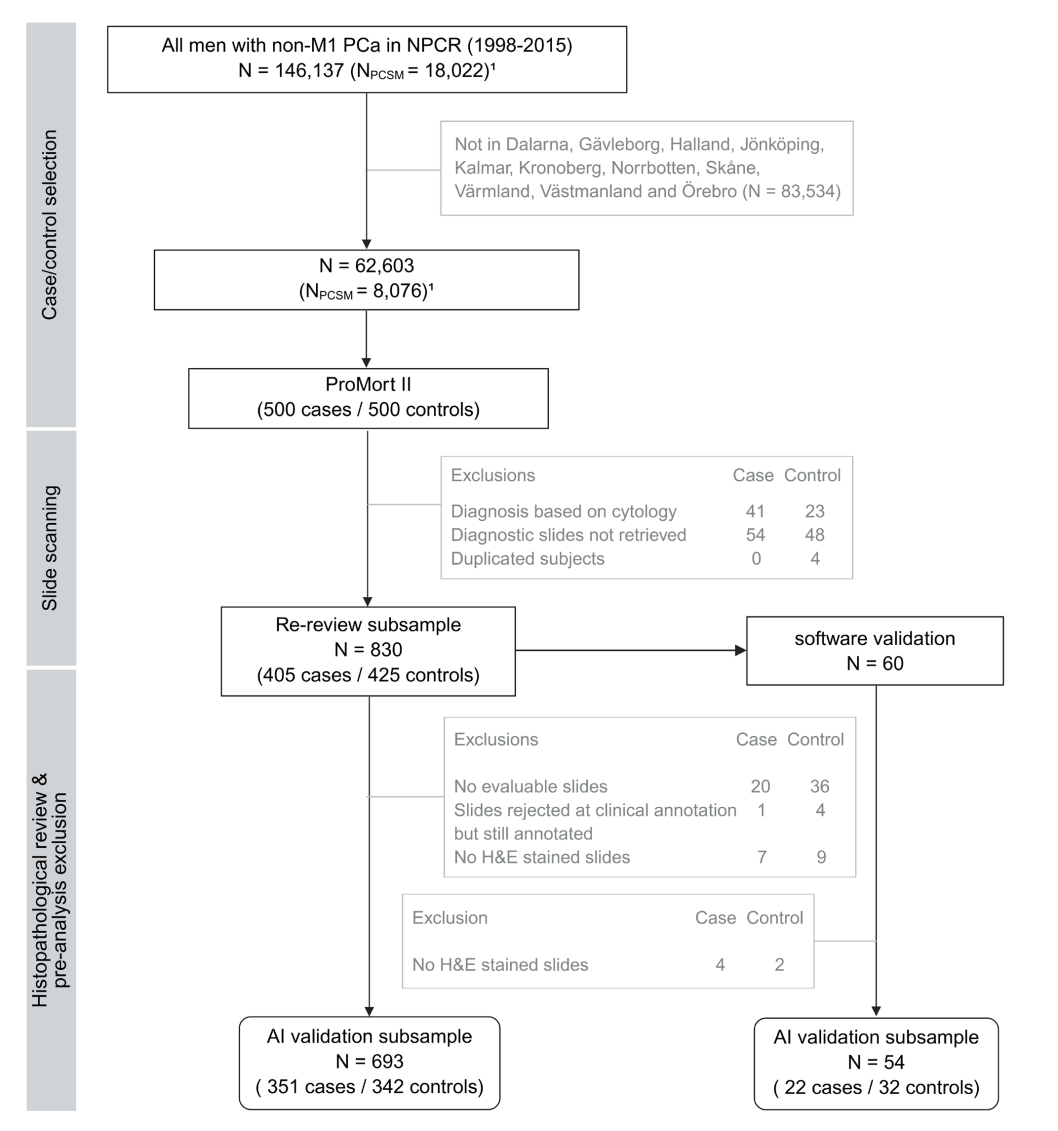
Flow chart of population selection for ProMort II sample, its re-review subsample and AI validation subsamples. N represents the number of patients. ¹Based on the data extracted from NPCR, 5 June 2020, but restricted to match conditions on 11 April 2017, when ProMort II was sampled. NPCR, National Prostate Cancer Register of Sweden; PCa, prostate cancer; PCSM, prostate cancer-specific mortality.

In total, 747 patients, of which 351 cases and 342 controls were in the case–control subsample and 22 cases and 32 controls were in the software validation subsample, were used for the AI model validation. Details of the ProMort II inclusion and exclusions are described in a flow chart in figure 2 and a summary of the population characteristics at the time of diagnosis is presented in table 2. Annotation protocols are provided in online supplemental material 2) (case–control subsample) and online supplemental material 3) (software validation subsample).

### Definition of reference standard

The reference standard consists of pathologists’ diagnoses at two levels: core-level and focus region-level. These annotations guide the evaluation of the AI system’s agreement with pathologists in diagnosing and grading prostate cancer in core needle biopsies. In this validation study, disagreements between pathologists are not resolved through consensus or majority vote. Instead, the primary reference standard consists of the annotations made by the principal reviewing pathologist (FG), who assessed the majority of cases in both ProMort I and ProMort II. When multiple pathologists annotated the same cores or focus regions, their assessments were retained alongside the primary reference and used in dedicated inter-observer analyses to quantify interobserver variability and to benchmark AI-pathologist agreement, as detailed in the Statistical Analyses section.

#### Core-level outcomes

Core-level outcomes refer to the review of the individual biopsy cores, that is, cylindrical tissue specimens, taken from each patient. The primary core-level outcomes are based on the pathologists’ annotations of the following features:

- Gleason score: The GS for a malignant individual biopsy core is determined by the most prevalent Gleason grade (primary grade) and the second most prevalent Gleason grade (secondary grade). However, in accordance with contemporary grading standards (2014 ISUP guidelines), any high-grade pattern (Gleason 4 or 5) was designated as the secondary pattern regardless of extent.
- Cancer length: The tumour extent of each core is measured as the total length of cancer, reported in millimetres. This measurement includes any intervening benign or non-invasive tissue, empty spaces between core fragments and all cancerous foci, regardless of their separation.

To ensure alignment across reviewers, cores within each slide were required to be annotated in a consistent order (left to right, top to bottom) so that core labels by different reviewers would generally correspond to each other.

#### Focus region-level outcomes

Focus regions refer to distinct tumour areas within malignant cores. For each identified focus region, pathologists annotated the high-risk features including:

- Cribriform pattern: The identification (presence or absence) of cribriform cancer morphology.
- PNI: The identification (presence or absence) of PNI.

To ensure alignment across reviewers, focus regions within each core were required to be annotated in a consistent order (left to right, top to bottom) so that region labels by different reviewers would generally correspond to each other. Minor inconsistencies may occur due to inter-observer variation in defining the boundaries of individual regions.

#### Spatial annotations

All core-level and focus region-level annotations are accompanied by corresponding spatial delineations on the WSIs. Each annotated region is demarcated by a closed polygon defined by a series of coordinates, enabling precise localisation of the annotated areas. For Gleason score 7 cores, grade 4 areas were additionally delineated to enable detailed pattern analysis. These spatial annotations ensure reproducible identification of the exact tissue regions assessed by the pathologists and enable comparison between pathologist and AI assessments on the same tissue areas.

### Preanalysis exclusion criteria

Prior to AI validation analyses, all WSIs underwent standardised screening and quality control to ensure data integrity and compatibility with the AI system. The exclusion criteria included:

- Slides not stained by H&E according to the staining information recorded during the histopathological review step. This criterion resulted in the exclusion of 11 cases and 9 controls from ProMort I Skåne and Örebro subsample; 7 cases and 9 controls from ProMort II case–control subsample; and 4 cases and 2 controls from ProMort II software validation subsample.
- Slides that were rejected during the pathologist clinical review but erroneously retained his/her diagnostic annotations in the database. This resulted in the exclusion of 1 case and 4 controls from the ProMort II case-control subsample.

Application of these exclusion criteria yielded the final AI validation datasets:

- ProMort I Skåne and Örebro subsample (N=290): 146 cases and 144 controls.
- ProMort II:
  - Case–control subsample (N=693): 351 cases and 342 controls.
  - Software validation subsample (N=54): 22 cases and 32 controls.

### Data independence

The population of ProMort I and II is fully external to the AI system, originating from different clinical sites and laboratories than those used during AI training, and scanned on a scanner not involved in the collection of training data. While there is some temporal overlap between the datasets (eg, between ProMort and the 2012–2015 STHLM3 cohort used for AI training), the geographic separation of the populations ensures that the training and testing data remain independent.

### Statistical analyses

#### Overview of statistical analyses

All primary and secondary analyses follow a three-part validation framework:

- Validation against the consistent reference standard.
- Subgroup analysis: Evaluate performance across different geographic regions.
- Sensitivity analyses:
  - Assess performance across multiple equally sized time intervals based on sample collection dates.
  - Assess performance against alternative reference standards, using subsets annotated by different pathologists

The framework above will be applied to:

1. **Primary analysis**: Cancer diagnosis and Gleason grading.
2. **Secondary analysis**: Cancer length prediction
3. **Secondary analysis**: Cribriform cancer detection.
4. **Secondary analysis**: PNI detection.

Analyses I, II and III A will be performed on samples for which the primary reference standard is available, defined as the annotations made by the principal reviewing pathologist (FG), who reviewed the majority of cases in every validation subsample. Analyses involving alternative reference standards and interobserver comparisons are described separately in Analysis III B (see tables 3 and 4 for details).

#### Details of statistical analyses

The AI system performance will be evaluated at the core level for cancer diagnosis, cancer grading and cancer length estimation. For detection of cribriform pattern and PNI, performance will be evaluated at core level using reference standards aggregated from focus region annotations. The cores in which the AI system preprocessing fails to detect any tissue will be reported and excluded from any subsequent analyses. Grading and cancer extent analyses will be performed using two approaches: (1) including all cores (benign and malignant) and (2) restricting to cores with malignant diagnoses by the reference pathologist. Cancer detection analysis will include all cores, while PNI and cribriform analyses will be performed on malignant cores only. To quantify uncertainty around all performance estimates, 95% CIs will be calculated using non-parametric bootstrap resampling clustered on patients (n=1000 iterations). The evaluation metrics are summarised in table 5.

**Table 5:** Summary of evaluation metrics for primary and secondary analyses

Outcome	Agreement	Discrimination	Calibration
**Cancer diagnosis**	Sensitivity, specificity Confusion matrix	AUC-ROC	Calibration slope and intercept Smoothed calibration plot
**Gleason grading**	Quadratic weighted Cohen’s kappa Linear weighted Cohen’s kappa Confusion matrix	Ordinal C-index	/
**Cancer length**	RMSE MAE Bland-Altman limits	Pearson correlation	Scatter plot with overlaid non-parametric smoother line
**Cribriform pattern**	Sensitivity, specificity Unweighted Cohen’s kappa Confusion matrix	AUC-ROC	Calibration slope and intercept Smoothed calibration plot
**Perineural invasion**	Sensitivity, specificity Unweighted Cohen’s kappa Confusion matrix	AUC-ROC	Calibration slope and intercept Smoothed calibration plot

Metrics are categorised by their evaluation purpose: agreement (concordance between AI predictions and reference standard), discrimination (ability to distinguish between outcome categories), and calibration (alignment between predicted probabilities and observed frequencies). All metrics will be reported with 95% CIs calculated using non-parametric bootstrap resampling (n=1000 iterations).

AI, artificial intelligence; AUC-ROC, area under the receiver operating characteristic curve; C-index, concordance index; MAE, mean absolute error; PPV, positive predictive value; RMSE, root mean squared error.

##### Primary analysis: diagnosis and Gleason grading

The concordance between the AI system’s outputs, including cancer diagnosis (positive/negative), GS and ISUP grade, and the corresponding reference standards will be quantified. Annotations by the pathologist FG will serve as the primary reference standard and cores annotated by other pathologists will be used only for sensitivity analysis. For the ProMort II software validation subsample, where FG performed annotations twice independently, the first round of annotations will serve as the reference standard, with the intraobserver agreement between the two rounds previously reported.^25^

###### Cancer diagnosis

For the binary classification between benign and malignant cores, we will calculate sensitivity (true positive rate) and specificity (true negative rate). The AI system’s cancer probability output will be evaluated using area under the receiver operating characteristic curve (AUC-ROC) to assess discriminative ability and using calibration plots to examine the alignment between predicted probability of outcome and observed outcomes.

Cancer diagnosisFor the binary classification between benign and malignant cores, we will calculate sensitivity (true positive rate) and specificity (true negative rate). The AI system’s cancer probability output will be evaluated using area under the receiver operating characteristic curve (AUC-ROC) to assess discriminative ability and using calibration plots to examine the alignment between predicted probability of outcome and observed outcomes.

###### Gleason score/ISUP grade

To quantify agreement between AI predictions and reference standard for the grading systems, we will employ quadratically weighted kappa (QWK) as the primary measure, with linearly weighted kappa and confusion matrices reported additionally. Ordinal concordance index (C-index) will be reported to measure model discrimination.^43^ For GS, patterns will be encoded as ordinal variables: benign (0), 3+3 (1), 3+4 (2), 4+3 (3), 3+5 (4), 4+4 (5), 5+3 (6), 4+5 (7), 5+4 (8), 5+5 (9). ISUP grades naturally form an ordinal scale (0–5), with grade 0 representing benign tissue.

- Subgroup analysis: evaluate performance across different geographic regions

Cancer diagnosis and grading performance metrics will be computed using geographical stratification, which will be conducted at the county level for all Swedish regions except Skåne. Given the large sample size from Skåne county, further stratification at the municipal level will be implemented for this region. This analysis accounts for potential batch effects arising from inter-institutional variability in slide preparation protocols, including tissue processing and staining procedures.

- Sensitivity analysis: evaluate performance across sample collection dates

Analysis will be stratified by sample collection date by grouping the cores into multiple bins of equal time duration. To ensure unbiased performance comparisons across time periods, stratified sampling will be employed within each temporal bin to achieve comparable ISUP grade distributions. This approach allows assessment of whether temporal factors, such as evolving laboratory practices or tissue preservation methods, influence model grading performance.

- Sensitivity analysis: assess performance against alternative reference standards

We will quantify all-against-all pairwise agreements in panels comprising pathologists and the AI system to establish whether AI versus reference standard discrepancies fall within the range of interobserver variation typically observed among pathologists.

- For the ProMort I Skåne and Örebro subsample, a subset of 548 cores was annotated by three pathologists (FG, LM, MF).
- For the ProMort II software validation subsample, a subset of 349 cores was annotated by three pathologists (FG, MF, OAs).

For both subsets, pairwise agreement calculations will be conducted, enabling six comparisons per subset (three AI-pathologist and three pathologist-pathologist). The average AI-pathologist agreement will be compared with the average pathologist-pathologist agreement to determine whether the AI system achieves expert-level performance.

### Secondary analysis: cancer length prediction

We will quantify the concordance between the AI system’s prediction of linear cancer extent (in millimetres) and the reference standards at the core level. Annotations by the pathologist FG will serve as the primary reference standard and cores annotated by other pathologists will only be used for sensitivity analysis.

- Performance metrics: Concordance will be assessed using root mean squared error (RMSE), mean absolute error (MAE) and Bland-Altman limits of agreement as the primary measures, with Pearson’s correlation coefficient reported additionally. Scatter plots will visualise the relationship between predicted and reference standard cancer lengths.
- Subgroup analysis: Geographical stratification will be conducted as described for the primary grading analysis, with RMSE, MAE, Bland-Altman limits and correlation coefficients computed separately for each region.
- Sensitivity analysis: Samples will be stratified by collection time as described for the primary grading analysis, with RMSE, MAE, Bland-Altman limits and correlation coefficients computed separately for each time period.
- Sensitivity analysis: Agreement between AI and multiple pathologists will be assessed and compared using the same structure as the grading sensitivity analysis, with RMSE, MAE, Bland-Altman limits and correlation coefficients computed quantifying pairwise concordance. The analysis will be applied to the 349 cores annotated by all three pathologists (FG, MF, OAs) within the ProMort II software validation subsample.

### Secondary analysis: cribriform cancer detection

Cribriform pattern detection performance will be evaluated following the same analytical framework as the primary grading analysis, with the following modifications:

- All analyses will be conducted at core level, using reference standards aggregated from focus region annotations.
- Performance metrics: As a binary classification task, the prediction probability outputs will be converted to binary predictions using a predetermined threshold of 0.5, which was optimised during model training through loss function weighting. Evaluation will include sensitivity, specificity, AUC-ROC, confusion matrices and calibration plot. Unweighted Cohen’s kappa will replace the weighted kappa measures for concordance assessment.
- Subgroup analysis: Geographical stratification will be conducted as described for the primary grading analysis, with sensitivity, specificity, confusion matrices and unweighted Cohen’s kappa computed separately for each region.
- Sensitivity analysis: Samples will be stratified by collection time as described for the primary grading analysis, with sensitivity, specificity, confusion matrices and unweighted Cohen’s kappa computed separately for each time period.
- Sensitivity analysis: Agreement between AI and multiple pathologists will be assessed and compared using the same structure as the grading sensitivity analysis, with unweighted kappa quantifying pairwise agreements.

- ProMort I: 288 cores annotated by all three pathologists (FG, LM, MF), and additional cores annotated by two pathologists: 193 cores by FG and LM and 266 cores by FG and MF.
- ProMort II: 196 cores annotated by all three pathologists (FG, MF, OAs).

Note that the AI system was developed to detect cribriform architecture regardless of its histological context—whether occurring within invasive acinar adenocarcinoma or intraductal carcinoma (IDC). This approach aligns with the 2019 ISUP consensus recommendations,^7^ which advocate for combined assessment of invasive and intraductal cribriform patterns for prognostic evaluation and treatment planning.

### Secondary analysis: PNI detection

PNI detection performance will be evaluated using an identical analytical framework as cribriform pattern detection. All analyses will be conducted at the core level with the same evaluation metrics (sensitivity, specificity, AUC-ROC, confusion matrices, calibration plot and unweighted Cohen’s kappa) using a 0.5 threshold for binary classification, subgroup analysis and sensitivity analyses approaches as described for cribriform detection above.

### Exploratory analysis

#### Prognostic stratification

As an exploratory assessment of whether AI-assigned grades preserve established prognostic patterns, we will conduct a patient-level survival analysis based on AI-derived Gleason scores and ISUP grades. For this purpose, all available biopsy WSIs from each patient will be processed jointly by the AI system by pooling all tissue-containing regions across slides into a single inference batch, thereby generating a patient-level grade. Patients for whom one or more WSIs were deemed inadequate for annotation by the pathologists (eg, poor quality of digitisation) will be excluded from this exploratory analysis to ensure that the AI model evaluates the complete set of biopsies for each patient. Kaplan-Meier curves for prostate cancer-specific mortality will be generated to evaluate whether the AI-assigned grade categories exhibit the expected ordering and separation of risk. Given the case-enriched design of the ProMort cohorts, these analyses will be interpreted exclusively as an assessment of relative stratification rather than as estimates of population-level absolute risk. The curves will not be compared with survival stratification based on pathologists’ grades, as pathologist annotations are available only at the core level, and patient-level aggregation based on subjective rules would introduce additional variability and reduce the comparability of the analysis.

#### Cost-effectiveness and workflow modelling

Although full health-economic evaluation lies outside the scope of this diagnostic validation, we will consider the potential for future decision-analytic or cost-effectiveness studies incorporating AI-assisted Gleason grading. Once validated, the diagnostic model may be integrated into established modelling frameworks–such as microsimulation or workflow simulation approaches similar to those used by Du *et al*^44^–to assess downstream clinical impact, workload implications and cost-effectiveness of adopting AI-assisted prostate cancer grading in routine practice.

### Analysis granularity considerations

For Gleason score assessment, the primary reference pathologist FG reviewed all cores on all slides for each assigned patient according to the review protocols (onlinesupplemental files 1^3^). However, we report only core-level outcomes rather than patient-level analyses. Since patient-level reference annotations were not collected, deriving patient-level outcomes would require applying post hoc aggregation rules (eg, taking the maximum/average/majority Gleason score across cores) to both reviewer annotations and AI predictions. Such aggregation approaches can introduce systematic bias or mask model performance limitations, particularly given that clinical practice involves variable and subjective approaches to patient-level scoring.^7^ Therefore, our concordance analyses focus on core-level AI-pathologist agreement as the most direct and unbiased assessment of model performance.

For focus-region annotations for PNI and cribriform cancer detection, analyses are performed at the core level for several methodological reasons. Focus-region level comparison is not feasible due to interobserver variability in spatial delineation, as pathologists may define different numbers and boundaries of focus regions within the same core based on varying interpretations of tissue gaps and morphological continuity. Slide-level and patient-level aggregations, while technically possible, would not provide meaningful analytical value beyond simple aggregation of core-level findings. Additionally, it is not uncommon for slides to contain only one core per slice, meaning core-level granularity could be used to approximate slide-level assessment. This allows the core-level validation results on ProMort to be compared with the AI model’s previous validation performance at slide level, providing important reference baselines for performance evaluation.

### Limitations and interpretive considerations

#### Generalisability limitations

The validation cohorts present spectrum and prevalence limitations that may affect generalisability. Both ProMort I and II use case–control designs with 50% cancer-related mortality, substantially higher than typical clinical populations, which may limit fair comparison of performance metrics with other AI validation studies using different cohort compositions. Additionally, ProMort I predominantly includes lower-grade cases (Gleason ≤7), creating spectrum bias that restricts assessment of AI performance across the full range of prostate cancer aggressiveness. These distributional characteristics should be considered when interpreting performance metrics or comparing results with other validation studies for AI-assisted cancer diagnostic systems. While subjects were excluded for various reasons, we specifically clarify two exclusion types that might be mistaken for sources of bias. Missing slide exclusions resulted from institutional slide retention policies (eg, discarding slides older than 10 years) rather than diagnostic-related factors. Exclusions due to inadequate slide quality during review were minimal and did not systematically target specific patient subgroups or tumour characteristics.

#### Information bias

Although pathologists were blinded to original Gleason scores and individual case-control status, awareness that both ProMort I and II case–control subsamples have higher mortality rates compared with unselected clinical populations may introduce unconscious grading bias. Pathologists might unconsciously assign higher grades when reviewing cases from cohorts known to be enriched for fatal outcomes, which could artificially increase or decrease AI-pathologist agreement depending on whether the AI exhibits systematic tendencies for overgrading or undergrading, respectively.

#### Interobserver analysis limitations

The ProMort I validation subsample employed a hierarchical annotation protocol where subsequent pathologist evaluations were contingent on prior assessments. The second reviewer, LM, evaluated only cores classified as malignant by FG, while MF assessed exclusively those cores exhibiting interobserver discordance between FG and LM. This design creates several methodological constraints that affect the interpretation of interobserver analyses:

- **Scope of analysis:** Interobserver comparisons are restricted to diagnostically challenging samples requiring adjudication rather than representing general diagnostic concordance across the full spectrum of cases.
- **Underestimation of pathologist concordance:** The exclusion of clear benign samples (where high agreement would be expected) leads to systematic underestimation of true pathologist-pathologist and pathologist-AI agreement compared with protocols evaluating complete sample sets, especially pronounced for FG versus LM concordance.
- **Comparative interpretation:** Results should be interpreted as evaluating whether AI performance on diagnostically challenging samples is comparable to interpathologist agreement on the same difficult samples, rather than as measures of overall diagnostic capability.

These constraints were predetermined by the original study design and cannot be modified retrospectively. ProMort II provides a more balanced inter-observer comparison framework for validation of these findings.

#### Measurement bias

For cribriform pattern detection, borderline cases were dichotomised as present/absent without an ‘equivocal’ category. This forced dichotomisation may underestimate true AI-pathologist agreement, as disagreements on genuinely ambiguous cases are counted as errors rather than recognised as inherent uncertainty in the reference standard.

#### Biopsy core segmentation errors

In ProMort II, the case–control subsample used automated tissue detection and core delineation, which occasionally partitioned a biological biopsy core into several tissue fragments. As reviewers’ annotations and AI model predictions are generated based on the identical delineated regions, the heterogeneity in granularity does not affect the fairness of AI-pathologist comparisons. However, this may impose higher demands on the AI system performance, as diagnostic errors have a greater impact when tissue segments contain limited morphological context and less redundancy than full-length biopsy cores.

## Ethics and dissemination

The study is conducted in agreement with the Declaration of Helsinki and approved by the Swedish Regional Ethics Review Board and the Swedish Ethical Review Authority (permits 2012/1586-31/1, 2016/613-31/2, 2019-01395, 2019-05220). Data were obtained from the NPCR of Sweden, which operates under the Swedish Patient Data Act (Patientdatalagen 2008:355, Chapter 7) and the EU General Data Protection Regulation (GDPR), without reliance on individual informed consent. The study results will be submitted for publication in an open-access format, regardless of whether the findings are positive, negative or inconclusive in relation to the study hypothesis.

## Study status

The key time points for this retrospective AI validation study are: (1) Confirmation of all AI model updates and acquisition of the final AI model version, (2) Establishment of the prespecified statistical analysis plan for validation data, (3) Conducting the final evaluation on validation data according to the prespecified plan. Respecting this timeline ensures no information leakage from the validation data influences the AI model design, and conversely, that validation analysis plans are not biased by prior knowledge of the latest model’s performance or limitations. The study status on this timeline is as follows:

1. 31 July 2025: All model updates were confirmed and the latest AI model version was obtained and locked for validation purposes.
2. 22 September 2025: The protocol covering AI model evaluation on PROMORT validation datasets was submitted to be made publicly available as a pre-print on medRxiv (https://www.medrxiv.org/content/10.1101/2025.09.22.25336169v1).
3. October 2025: Final evaluation of the AI model on the PROMORT validation datasets for cancer detection and Gleason grading will be conducted according to the prespecified analysis plan with results to be published in a peer-reviewed journal.

## Supplementary material

10.1136/bmjopen-2025-111361 online supplemental file 1

10.1136/bmjopen-2025-111361 online supplemental file 2

10.1136/bmjopen-2025-111361 online supplemental file 3
