# Supplementary material for: Retrospective validation of an artificial intelligence system for diagnostic assessment of prostate biopsies on the ProMort cohort: study protocol
Source: BMJ Open. 2025 Dec 24;15(12):e111361. doi: 10.1136/bmjopen-2025-111361 (PMC12742080; doi:10.1136/bmjopen-2025-111361)
Supplement: Supplementary file 4 [file bmjopen-2025-111361.reviewer_comments.pdf]

PEER REVIEW HISTORY

BMJ Open publishes all reviews undertaken for accepted manuscripts. Reviewers are asked to complete a checklist review form and are provided with free text boxes to elaborate on their assessment. These free text comments are reproduced below.

ARTICLE DETAILS

Title (Provisional)

Retrospective Validation of an Artificial Intelligence System for Diagnostic Assessment of Prostate Biopsies on the ProMort Cohort: Study Protocol

Authors

Ji, Xiaoyi; Zelic, Renata; Aspegren, Oskar; Mulliqi, Nita; Fiorentino, Michelangelo; Giunchi, Francesca; Molinaro, Luca; Boman, Sol Erika; Szolnoky, Kelvin; Liu, Luana Xuan; Pettersson, Andreas; Vincent, Per Henrik; Eklund, Martin; Akre, Olof; Kartasalo, Kimmo

---

VERSION 1 - REVIEW

**Reviewer**	**1**
**Name**	**Mai, Zhipeng**
**Affiliation**	**Peking Union Medical College Hospital**
**Date**	**12-Nov-2025**
**COI**	**None**

This article presents a rigorously designed and transparently reported study protocol for the retrospective validation of an AI system for prostate biopsy diagnosis using the Swedish ProMort cohort. The study comprehensively evaluates AI performance in Gleason grading, cancer length measurement, cribriform pattern, and perineural invasion detection on fully independent external datasets, adhering to CLAIM and STARD-AI guidelines, which demonstrates high scientific rigor and reproducibility.

Suggestions for Improvement:

1. In the "Artificial intelligence system" section, provide more explicit details on the specific AI model architecture, training data sources, sample size, data preprocessing methods, and hyperparameter settings.

2. In the "Definition of reference standard" section, elaborate on how disagreements between pathologists were resolved (e.g., majority vote, expert adjudication).

3. Beyond statistical agreement (e.g., Kappa, AUC), consider adding a simulated analysis of clinical impact, such as concordance between AI and pathologists on treatment decisions (e.g., active surveillance vs. curative treatment).

VERSION 1 - AUTHOR RESPONSE

- Dr. Zhipeng Mai, Peking Union Medical College Hospital

Comments to the Author:

This article presents a rigorously designed and transparently reported study protocol for the retrospective validation of an AI system for prostate biopsy diagnosis using the Swedish ProMort cohort. The study comprehensively evaluates AI performance in Gleason grading, cancer length measurement, cribriform pattern, and perineural invasion detection on fully independent external datasets, adhering to CLAIM and STARD-AI guidelines, which demonstrates high scientific rigor and reproducibility.

Suggestions for Improvement:

1. In the "Artificial intelligence system" section, provide more explicit details on the specific AI model architecture, training data sources, sample size, data preprocessing methods, and hyperparameter settings.

A: To improve clarity for readers who may not fully go through the original development and validation publications, we have added a concise description of the AI system's training data, preprocessing pipeline, and core architectural components in the *Artificial intelligence system* section. These additions summarise the key aspects relevant for understanding the system to be validated in this study, while more detailed specifications remain available in the original protocol-based development papers[1,2]. The manuscript has been updated accordingly.

1. Mulliqi N, Blilie A, Ji X, Szolnoky K, Olsson H, Titus M, et al. Development and retrospective validation of an artificial intelligence system for diagnostic assessment of prostate biopsies: study protocol. BMJ Open. 2025 July 7;15(7):e097591.
2. Mulliqi N, Blilie A, Ji X, Szolnoky K, Olsson H, Boman SE, et al. Foundation models -- A panacea for artificial intelligence in pathology? [Internet]. arXiv [cs.CV]. 2025. Available from: http://dx.doi.org/10.48550/ARXIV.2502.21264

2. In the "Definition of reference standard" section, elaborate on how disagreements between pathologists were resolved (e.g., majority vote, expert adjudication).

A: We thank the reviewer for highlighting this point. To clarify, in this validation study, disagreements between pathologists are not merged into a single consensus or adjudicated outcome. Instead, we follow a predefined strategy in which the primary reference standard is given by the annotations of the principal reviewing pathologist (F.G.), who evaluated the majority of cases. For subsets of samples reviewed by additional pathologists, each set of

annotations is retained separately and used as an alternative reference standard in analyses assessing inter-observer variability and comparing AI-pathologist agreement with the range of pathologist–pathologist agreement. We have revised the *Definition of reference standard* section to make this approach clearer and to link it explicitly to the *Statistical Analyses*.

3. Beyond statistical agreement (e.g., Kappa, AUC), consider adding a simulated analysis of clinical impact, such as concordance between AI and pathologists on treatment decisions (e.g., active surveillance vs. curative treatment).

A: We appreciate the reviewer's thoughtful suggestion and agree that understanding the potential clinical implications of AI-assisted grading is of considerable interest. In the present study, however, the AI system under evaluation is a task-specific diagnostic model developed exclusively for assigning Gleason scores and selected histological features. The model was neither trained nor intended to estimate patient prognosis or recommend management strategies. Because treatment decisions for prostate cancer rely on a combination of clinical, pathological, imaging, and patient-specific factors, a simulation of treatment selection (e.g., active surveillance vs. curative therapy) cannot be meaningfully performed using outputs from this diagnostic-only system.

To nevertheless provide insight into the potential relevance of AI-derived grades for downstream clinical research, we will incorporate an exploratory analysis evaluating whether AI-assigned ISUP grades and Gleason scores preserve expected prognostic signatures within the available data. While the ProMort cohorts are not population-based and therefore do not allow interpretation of absolute survival probabilities without appropriate re-weighting, relative separation between grade categories can still illustrate whether the model captures morphological signals associated with established risk gradients. We do not plan to compare these curves directly with survival stratification based on pathologists' grades, as (i) the case-enriched design limits interpretability of absolute risk comparisons across annotators, and (ii) pathologist assessments are available only at the core level and require aggregation rules that would introduce additional variability and compromise the comparability of patient-level prognostic analyses.

Importantly, beyond the scope of this diagnostic validation, our group is actively developing dedicated prognostic AI models that integrate additional clinical and pathological variables and are explicitly designed to estimate outcome risk and support treatment-related decision modelling. These future works will be capable of addressing the type of clinical impact analyses suggested by the reviewer in a methodologically appropriate manner, and their design and validation will be detailed in separate, dedicated future study protocols.

Finally, clinical utility can also be evaluated through decision-analytic or cost-effectiveness modelling. While such analyses lie outside the objectives of the current diagnostic validation, we will note in the protocol that complementary modelling approaches (e.g., the framework used by Du et al. 2025[1]) represent important future avenues for assessing the broader clinical impact of AI-assisted pathology.

An *Exploratory analyses* subsection has been added to the *Statistical Analyses* section describing the planned prognostic-stratification assessment and outlining the potential for future cost-effectiveness modelling.

1. Du X, Hao S, Olsson H, Kartasalo K, Mulliqi N, Rai B, et al. Effectiveness and Cost-effectiveness of Artificial Intelligence-assisted Pathology for Prostate Cancer Diagnosis in Sweden: A Microsimulation Study. Eur Urol Oncol. 2025 Feb;8(1):80–6.

- Extra response:
  In addition to addressing the reviewers' comments, we made one minor clarifying revision to the manuscript regarding the delineation process used in the ProMort II case-control subsample. After the initial submission, we recognised that the protocol did not explicitly describe that tissue detection and segmentation in this subsample were generated by the automated segmentation component of the assessment platform, which occasionally resulted in fragmentation of individual biopsy cores. This clarification does not alter the study design, reference standard definition, or planned analyses, as both pathologist annotations and AI predictions are performed on the same spatial regions. However, we considered it important to document this procedural detail for completeness and transparency. Accordingly, we have added a brief description to the *Data sources-ProMort II* section and included a short note under *Limitations and interpretive considerations* to highlight this granularity issue.
